# Prevalence, Symptoms, and Associated Risk Factors for Depressive Symptoms Among Undergraduate Students of Non-Medical Universities in Mwanza, Tanzania

**DOI:** 10.3390/diseases13080268

**Published:** 2025-08-19

**Authors:** Stanley Mwita, Mathew Ouma, Warren Edwin, Deogratias Katabalo, Karol Marwa

**Affiliations:** 1 Department of Pharmaceutics and Pharmacy Practice, Catholic University of Health and Allied Sciences, Mwanza 33102, Tanzania; 2 Department of Pharmacology, Catholic University of Health and Allied Sciences, Mwanza 33102, Tanzania

**Keywords:** prevalence, symptoms, risk factors, depression, students

## Abstract

Background: University students are vulnerable to depression due to the transitional nature of their life stage, which often involves increased academic pressures and social changes. This study aims to examine the prevalence, symptoms, and associated risk factors for depressive symptoms among undergraduate students at non-medical universities. Methods: This cross-sectional study was conducted at non-medical universities in Mwanza Region, Tanzania. A self-administered, structured questionnaire was used to collect the data. The presence and severity of depressive symptoms were assessed using the Beck Depression Inventory (BDI-II). Results: A total of 768 students participated in the study. The prevalence of depressive symptoms was 35.7%. A significant proportion experienced loss of interest and pleasure (*n* = 516; 67.2%), felt easily tired (*n* = 373; 48.6%), and had difficulty making decisions (*n* = 303; 39.4%). A significant relationship was observed between age and depressive symptoms, with participants aged 25 and above reporting higher rates of depressive symptoms (53.2%) compared to those aged 18–24 (28.8%) (*p* < 0.001). Similarly, the year of study was significantly associated with depressive symptoms; fourth-year students had the highest proportion of depressive symptoms (64.3%), while first-year students had the lowest proportion (26.2%) (*p* < 0.001). Conclusion: This study found that over one-third of undergraduate students in non-medical universities suffer from depressive symptoms. This high prevalence rate highlights an urgent need for targeted mental health interventions within university settings to prevent long-term academic, social, and psychological consequences. Campus counseling services should prioritize screening for key symptoms while developing stress-management programs tailored to academic progression challenges.

## 1. Introduction

Depression, also known as depressive disorder, is characterized by persistent psychological experiences of loss, sadness, and hopelessness in an individual’s life [1]. Depression is a significant public health concern globally, particularly among young adults and students. An estimated 3.8% of the population experiences depression, and about 280 million people in the world have depression [2]. In low- and middle-income countries (LMICs), including Tanzania, depression among university students is often underdiagnosed and undertreated due to limited access to campus-based mental health professionals, stigma, and lack of awareness [3,4]. University students are particularly vulnerable due to the transitional nature of their life stage, which often involves increased academic pressures, social changes, and the need for greater support [5,6].

The prevalence of depression among university students varies widely across different regions and cultures. A previous systematic review reported that the pooled prevalence of depression among college students was 33.6%. The highest prevalence of depression symptoms was found in the African region, 40.1%, in LMICs and among medical college students [7]. During a depressive episode, a person experiences a depressed mood (feeling sad, irritable, or empty). They may feel a loss of pleasure or interest in activities. Other symptoms may include poor concentration, feelings of excessive guilt or low self-worth, hopelessness about the future, thoughts about dying or suicide, disrupted sleep, changes in appetite or weight, and feeling very tired or low in energy [2,8].

Research has shown that various risk factors contribute to the development of depression among university students. These factors may include academic stress, personality traits, and prior mental health history. Social factors, such as peer relationships and family support, also play a significant role in students’ mental health [9]. Environmental factors, such as financial stress and living conditions, further complicate the mental health of students [10]. In Tanzania, previous studies have highlighted the mental health challenges faced by university students. Factors independently associated with depression included the year of study, substance abuse, unhappy interpersonal relationships, and chronic psychological or physical illness. Protective factors identified included residing off-campus and the perceived availability of social support, while risk factors encompassed a family history of mental illness and decreased academic performance [11,12]. Medical students are exposed to health-related curricula and mental health awareness that may influence both their risk and recognition of depressive symptoms. In contrast, non-medical students may lack similar exposure, coping strategies, or access to tailored mental health resources [13].

Despite these insights, there is a paucity of research focusing on non-medical universities in Tanzania, particularly in regions like Mwanza. This study aims to fill this gap by examining the prevalence, symptoms, and associated risk factors for depressive symptoms among undergraduate students at non-medical universities in Mwanza, Tanzania.

## 2. Materials and Methods

### 2.1. Study Design and Setting

This cross-sectional study was conducted from April to May 2022 at Saint Augustine University (SAUT) and the College of Business Education (CBE) in Mwanza Region, Tanzania. SAUT is a prominent private university affiliated with the Tanzania Episcopal Conference, offering various humanities, social sciences, business, and law academic programs. It has a diverse student population and is known for its emphasis on ethical leadership and social responsibility. Conversely, CBE is a public institution under the Ministry of Industry and Trade, specializing in business-related disciplines such as marketing, procurement, accounting, and information technology. Both institutions serve a large number of undergraduate students from various socio-economic backgrounds and regions across Tanzania. Although the study took place in May 2022 during the COVID-19 pandemic, no lockdowns or significant academic disruptions were in place at the two universities during the data collection period.

### 2.2. Study Population and Sample Size

The study population comprised undergraduate students. Students who were academically active and present on the campus during the data collection period were included in the study, whereas students who were severely ill were excluded. Severely ill students were identified based on visible signs of physical incapacity and self-declaration. The sample size was calculated using the Kish and Leslie formula, incorporating a 95% confidence level (corresponding to a standard normal value of 1.96), a 5% margin of error, and an estimated prevalence of mental distress among undergraduate students at the University of Gondar, Ethiopia (40.9%) [14]. This study was chosen due to contextual and regional similarities, including comparable socio-economic environments and university systems. This resulted in an initial sample size of 277 participants. Given that the study was conducted across two universities, this figure was doubled to ensure adequate representation, resulting in a final minimum sample size of 742.

For participant recruitment, the study employed a snowball sampling technique, a non-probability method particularly effective for reaching populations that may otherwise be difficult to access. This approach involved enlisting the assistance of initial participants to identify and refer other eligible individuals, thereby expanding the sample in a cascading manner.

### 2.3. Data Collection

A self-administered, structured questionnaire was used to collect information. Social, economic, and sociodemographic factors were included in the questionnaire. The presence and severity of depressive symptoms were assessed using the Beck Depression Inventory (BDI-II). Cronbach’s alpha coefficient for the BDI-II in our study was 0.87, indicating good internal consistency. The twenty-one items, which consisted of four statements concerning a specific depressive symptom grouped by increasing severity, were rated on a scale of 0 to 3 [15]. The overall score increased from 0 to 63. BDI scores of 14 or higher were categorized as the presence of depression [16,17]. According to BDI-II, a score of 0 to 4 is normal, 5 to 13 is borderline clinical depression, 14 to 19 is mild depression, 20 to 28 is moderate depression, and 29 to 63 is severe depression [15].

### 2.4. Data Analysis

The collected data were cleaned, coded, and entered into STATA Version 15 for analysis. Descriptive statistics (medians, interquartile ranges (IQRs), percentages, frequencies, and standard deviations) were used to summarize the continuous and categorical variables as appropriate. Chi-square tests were conducted to determine the relationship between categorical variables. To examine factors associated with depressive symptoms, a logistic regression analysis was performed. All factors in the bivariate analysis were included in the final model to ensure comprehensive adjustment for potential confounders. Data are presented as the crude odds ratio (COR) and adjusted odds ratio (AOR) with 95% confidence intervals as appropriate. Factors with a *p*-value of less than 0.05 were considered statistically significant.

### 2.5. Ethical Considerations

Ethical clearance was obtained from the joint CUHAS/BMC Ethics and Review Committee (2310/2022 and 2263/2022), approved on 28 April and 7 May 2022. Permission to conduct the study was sought from the Vice Chancellor of SAUT and CBE. Written informed consent was requested from all study participants. To ensure confidentiality, unique identification numbers were used instead of names.

## 3. Results

### 3.1. Sociodemographic Characteristics

A total of 768 students participated in the study. The majority were female, comprising 423 respondents (55.1%). The median age was 23 years, with an interquartile range of 21 to 25 years; the majority were aged between 18 and 24 (552; 71.9%). Most participants were in their third year of study, accounting for 329 individuals (42.8%). Additionally, a significant proportion reported having a good relationship with their parents (480; 62.5%), and nearly half described their family’s economic status as moderate (360; 46.9%). (Table 1).

### 3.2. Prevalence and Common Symptoms of Depression

The prevalence of depression among 768 students was 35.7%. Table 2 presents the various symptoms of clinical depression among 768 study participants. A significant proportion experienced loss of interest and pleasure (*n* = 516; 67.2%), felt easily tired (*n* = 373; 48.6%), had difficulty making decisions (*n* = 303; 39.4%), decreased appetite (*n* = 302; 39.3%), sleep disturbances (*n* = 296; 38.5%), and low mood (*n* = 299; 38.0%). The mean BDI score was 12.9 (SD ± 9.1). Most participants (64.3%) scored in the normal range (0–4), while the rest fell into borderline (10.6%), mild (11.1%), moderate (9.2%), or severe (4.8%) categories.

### 3.3. Factors Associated with Depressive Symptoms

Table 3 presents the influence of demographic and socio-economic factors on depression among the study participants. A significant relationship was observed between age and depression, with participants aged 25 and above reporting higher rates of depression (53.2%) compared to those aged 18–24 (28.8%) (*p* < 0.001). Similarly, the year of study was significantly associated with depression; fourth-year students had the highest proportion of depression (64.3%), while first-year students had the lowest proportion (26.2%) (*p* < 0.001). No significant associations were found between depression and gender (*p* = 0.472), parents’ relationship status (*p* = 0.201), or family economic status (*p* = 0.586).

Table 4 outlines both bivariate (COR) and multivariate (AOR) logistic regression analyses, identifying factors associated with depression. Age and year of study were significantly associated with depression in both analyses. Participants aged 25 and above had over twice the odds of experiencing depression compared to those aged 18–24 (AOR = 2.54, 95% CI: 1.79–3.62, *p* < 0.001). Regarding the academic year, fourth-year students showed a markedly increased risk (AOR = 4.06, 95% CI: 1.90–8.67, *p* < 0.001), and third-year students also had significantly higher odds (AOR = 1.55, 95% CI: 1.01–2.39, *p* = 0.047) compared to first-year students. Gender, parents’ relationship quality, and family economic status were not significantly associated with depression in the multivariate analysis.

## 4. Discussion

Depression among university students has become increasingly recognized as a global public health concern, with young adults experiencing a disproportionate burden due to academic, social, and economic pressures. The findings of this study reveal a substantial prevalence of depressive symptoms (35.7%) among undergraduate students from non-medical universities in Mwanza, Tanzania. About 14.0% of participants scored above 19 on the BDI-II scale, falling into the moderate (9.2%) and severe (4.8%) categories of depressive symptomatology. This subset represents individuals with clinically significant levels of depressive symptoms who may require immediate psychological evaluation and intervention since they are within a university context, where academic pressures, social transitions, and inadequate mental health support systems may exacerbate psychological distress. In contrast, previous studies conducted in Tanzania and Ethiopia reported lower prevalence rates, ranging from 14.0% to 28.2% [11,12,18]. Conversely, research from Ethiopia and India has highlighted even more alarming figures, with prevalence rates rapidly increasing to 45.9% [19] and 59.8% [20], respectively. The substantial prevalence of depressive symptoms among undergraduate students indicates a need for increased awareness and understanding of mental health issues within university settings. Differences in the reported prevalences might be due to variations in cultural attitudes towards mental health in different regions; differences in sample size, demographic characteristics, and academic disciplines among studies; and variations in research methodologies, including the tools used for diagnosing depression, the definitions of depression employed, and the timing of data collection. Cultural attitudes significantly influence help-seeking behavior for mental health issues, often leading to stigma and reluctance to seek professional assistance. Societal perceptions of mental illness as a sign of weakness or a personal failing can deter individuals from acknowledging their struggles.

The most commonly reported symptoms were loss of interest and pleasure (67.2%), fatigue (48.6%), difficulty making decisions (39.4%), sleep disturbances (38.5%), and low mood (38.0%). These findings are in line with the results reported in previous studies [12,21,22,23]. Loss of interest may correlate with disengagement from academic activities, affecting motivation and performance. The high prevalence of fatigue may be linked to academic workload, irregular sleep patterns, and lifestyle factors such as poor nutrition and prolonged screen time. Low mood has the potential to overlap with fatigue and may manifest in the form of emotional exhaustion or reduced motivation. There is a need for customized counseling and therapy programs to address these symptoms. For example, cognitive-behavioral therapy (CBT) can be tailored to address anhedonia (a loss of interest) and indecisiveness, while interventions for fatigue and sleep disturbances might include behavioral activation, sleep hygiene programs, and academic scheduling adjustments [24,25,26,27]. The presence of thoughts of ending life in 21.9% of participants is particularly alarming and hihlights the urgent need for mental health initiatives such as peer support groups and suicide prevention strategies in Tanzanian universities.

In this study, age emerged as a significant predictor of depressive symptoms, with students aged 25 years and older showing markedly higher rates (53.2%) compared to those aged 18–24 (28.8%). Logistic regression analysis confirmed that older students were more than twice as likely to experience depressive symptoms. This could be attributed to increased responsibilities, financial stress, and job market anxiety. However, this is inconsistent with findings from a similar study conducted in northern Tanzania [12], where age was not associated with depressive symptoms. The year of study also demonstrated a strong association with depressive symptoms. Fourth-year students had the highest odds of depressive symptoms, followed by third-year students, relative to first-year students. Similar patterns have been observed in a study conducted in the Kilimanjaro region of Tanzania. The increasing burden of academic workload, career uncertainties, and thesis requirements may contribute to this trend [28]. To address these academic progression challenges, implementing specific support programs for final-year students, such as mentorship initiatives, stress management workshops, and peer support groups, could provide practical assistance and enhance their well-being during this critical transition period.

This study did not observe a statistically significant difference between male and female students. This is in agreement with a previous study conducted among undergraduate medical students [12]. While females reported slightly lower rates (34.6%) than males (37.1%), the adjusted analysis suggested that gender was not an independent predictor of depressive symptoms. These findings could be attributed to the fact that both genders in this context are exposed to similar academic pressures, financial stress, and limited coping resources. Students with good parental and family economic status reported slightly lower rates of depressive symptoms; this is in line with previous studies conducted in Ethiopia and Tanzania [11,18]. Neither the quality of the parental relationship nor perceived family economic status showed a significant association with depressive symptoms in the multivariate model. However, the self-perceived family economic status may not fully reflect the financial burden. This result may indicate that gender, parental relationships, and perceived family economic status do not independently predict depressive symptoms, reinforcing the idea that socio-environmental, academic stress, peer relationships, or psychological factors may be more influential in determining the risk of depression among students [29,30].

This study provides valuable data on a relatively understudied population of non-medical undergraduate university students in Mwanza using a robust sample size and standardized diagnostic criteria. These findings may not apply to postgraduates, who may face different stress profiles. However, it is not without its limitations. The use of self-reported data raises the risk of social desirability and recollection biases, and the cross-sectional design prevents causal inference. Participants may underreport symptoms like suicidal thoughts or fatigue due to stigma, affecting validity. Additionally, the study did not assess potential confounders such as substance use, romantic relationships, or exposure to trauma, which could influence depression risk. Lastly, the use of snowball sampling could overrepresent certain social networks or peer groups.

Future studies should adopt longitudinal designs to explore the trajectory of depressive symptoms over time and the long-term outcomes of affected students. Qualitative research, including focus group discussions and in-depth interviews, may also provide richer insights into the lived experiences and coping mechanisms of depressed students. Furthermore, expanding research to include medical students, private universities, and postgraduates would help provide a more comprehensive picture of student mental health across educational sectors.

## 5. Conclusions

This study found that over one-third of undergraduate students in non-medical universities suffer from depressive symptoms, with symptoms such as loss of interest and pleasure, fatigue, difficulty making decisions, and sleep disturbances being particularly common. This high prevalence rate highlights an urgent need for targeted mental health interventions within university settings to prevent long-term academic, social, and psychological consequences. Age and year of study were significantly associated with depressive symptoms. Campus counseling services should prioritize screening for key symptoms while developing stress-management programs tailored to academic progression challenges. Additionally, university-wide mental health awareness campaigns could encourage early help-seeking behavior.

## Figures and Tables

**Table 1:** The demographic characteristics of the participants (*N* = 768).

Variable		Frequency	Percentage
Age (Years)	(Median ± IQR)	23 (21–25)	
	18–24	552	71.9
	25+	216	28.1
Sex	Male	345	44.9
	Female	423	55.1
Year of study	1	164	21.4
	2	233	30.3
	3	329	42.8
	4	42	5.5
Parent’s Relationship	Good	480	62.5
	Moderate	201	26.2
	Poor	87	11.3
Family Economic Status	Good	264	34.4
	Moderate	360	46.9
	Poor	144	18.7

**Table 2:** The prevalence and common symptoms of depression among the study participants (*N* = 768).

Symptom	Frequency	Percentage
**Psychological**		
Low mood	299	38.0
Loss of interest and pleasure	516	67.2
Feelings of guilt and a sense of worthlessness	172	22.4
Crying more than usual	252	32.8
Reduced self-esteem and confidence	225	29.3
Pessimistic thoughts about the future	228	29.7
Thoughts of ending life	168	21.9
**Physical**		
Sleep disturbances	296	38.5
Decreased appetite	302	39.3
Easily tired	373	48.6
**Cognitive**		
Difficulties in making decisions	303	39.4
Other symptoms	182	23.7
**Depression**		
Yes	274	35.7
No	494	64.3
**BDI values, Mean (SD)**	12.9 ± 9.1	
0 to 4 (normal)	494	64.3
5 to 13 (borderline)	81	10.6
14 to 19 (mild)	85	11.1
20 to 28 (moderate)	71	9.2
29 to 63 (severe)	37	4.8

**Table 3:** The factors influencing depressive symptoms among the study participants (*N* = 768).

Variables		Depression		* *p*-Value
		Yes, *n* (%)	No, *n* (%)	
Age (Years)	18–24	159 (28.8)	393 (71.2)	<0.001
	25+	115 (53.2)	101 (46.8)	
Gender	Male	128 (37.1)	217 (62.9)	0.472
	Female	146 (34.6)	277 (65.4)	
Year of study	1	43 (26.2)	121 (73.8)	<0.001
	2	66 (28.3)	167 (71.7)	
	3	138 (41.9)	191 (58.1)	
	4	27 (64.3)	15 (35.7)	
Parent’s relationship	Good	160 (33.3)	320 (66.7)	0.201
	Moderate	79 (39.3)	122 (60.7)	
	Poor	35 (40.2)	52 (59.8)	
Family economic status	Good	88 (33.3)	176 (66.7)	0.586
	Moderate	134 (37.2)	226 (62.8)	
	Poor	52 (36.1)	92 (63.9)	

* Chi-square test.

**Table 4:** Bivariate and multivariate analysis of factors associated with depressive symptoms among participants.

Variables	COR	*p*-Value	AOR	*p*-Value
Age (Years)				
18–24	1		1	
25+	2.81 (2.03–3.89)	<0.001	2.54 (1.79–3.62)	<0.001
Gender				
Female	1		1	
Male	1.11 (0.83–1.51)	0.457	1.29 (0.94 1.76)	0.116
Year of study				
1	1		1	
2	2.54 (1.79–3.62)	0.643	0.95 (0.60–1.51)	0.837
3	2.01 (1.33–3.03)	0.001	1.55 (1.01–2.39)	0.047
4	5.42 (2.61–11.29)	<0.001	4.06 (1.90–8.67)	<0.001
Parent’s relationship				
Good	1			
Moderate	1.29 (0.92–1.82)	0.137	1.15 (0.79–1.68)	0.467
Poor	1.35 (0.84–2.15)	0.214	1.27 (0.76–2.14)	0.360
Family economic status				
Good	1			
Moderate	1.18 (0.85–1.65)	0.316	0.93 (0.65–1.34)	0.704
Poor	1.13 (0.74–1.73)	0.572	0.79 (0.48–1.28)	0.335

## Data Availability

The data presented in this study are available upon request from the corresponding author.
